# Endothelium-Based Biomarkers Are Associated with Cerebral Malaria in Malawian Children: A Retrospective Case-Control Study

**DOI:** 10.1371/journal.pone.0015291

**Published:** 2010-12-29

**Authors:** Andrea L. Conroy, Happy Phiri, Michael Hawkes, Simon Glover, Mac Mallewa, Karl B. Seydel, Terrie E. Taylor, Malcolm E. Molyneux, Kevin C. Kain

**Affiliations:** 1 Sandra A. Rotman Laboratories, McLaughlin-Rotman Centre for Global Health, University Health Network-Toronto General Hospital, University of Toronto, Toronto, Canada; 2 Malawi-Liverpool-Wellcome Trust Clinical Research Programme, Blantyre, Malawi; 3 College of Medicine, University of Malawi, Blantyre, Malawi; 4 School of Tropical Medicine, University of Liverpool, Liverpool, United Kingdom; 5 Blantyre Malaria Project, College of Medicine, University of Malawi, Blantyre, Malawi; 6 Department of Internal Medicine, College of Osteopathic Medicine, Michigan State University, East Lansing, Michigan, United States of America; 7 Tropical Disease Unit, Division of Infectious Diseases, Department of Medicine, University of Toronto, Toronto, Canada; KEMRI/Wellcome Trust Research Programme, Kenya

## Abstract

**Background:**

Differentiating cerebral malaria (CM) from other causes of serious illness in African children is problematic, owing to the non-specific nature of the clinical presentation and the high prevalence of incidental parasitaemia. CM is associated with endothelial activation. In this study we tested the hypothesis that endothelium-derived biomarkers are associated with the pathophysiology of severe malaria and may help identify children with CM.

**Methods and Findings:**

Plasma samples were tested from children recruited with uncomplicated malaria (UM; n = 32), cerebral malaria with retinopathy (CM-R; n = 38), clinically defined CM without retinopathy (CM-N; n = 29), or non-malaria febrile illness with decreased consciousness (CNS; n = 24). Admission levels of angiopoietin-2 (Ang-2), Ang-1, soluble Tie-2 (sTie-2), von Willebrand factor (VWF), its propeptide (VWFpp), vascular endothelial growth factor (VEGF), soluble ICAM-1 (sICAM-1) and interferon-inducible protein 10 (IP-10) were measured by ELISA. Children with CM-R had significantly higher median levels of Ang-2, Ang-2:Ang-1, sTie-2, VWFpp and sICAM-1 compared to children with CM-N. Children with CM-R had significantly lower median levels of Ang-1 and higher median concentrations of Ang-2:Ang-1, sTie-2, VWF, VWFpp, VEGF and sICAM-1 compared to UM, and significantly lower median levels of Ang-1 and higher median levels of Ang-2, Ang-2:Ang-1, VWF and VWFpp compared to children with fever and altered consciousness due to other causes. Ang-1 was the best discriminator between UM and CM-R and between CNS and CM-R (areas under the ROC curve of 0.96 and 0.93, respectively). A comparison of biomarker levels in CM-R between admission and recovery showed uniform increases in Ang-1 levels, suggesting this biomarker may have utility in monitoring clinical response.

**Conclusions:**

These results suggest that endothelial proteins are informative biomarkers of malarial disease severity. These results require validation in prospective studies to confirm that this group of biomarkers improves the diagnostic accuracy of CM from similar conditions causing fever and altered consciousness.

## Introduction

Differentiating cerebral malaria (CM) from other conditions causing fever and altered consciousness is a clinical challenge, owing to the non-specific clinical presentations of CM (fever, coma, convulsions) and the high prevalence of incidental parasitaemia in malaria-endemic areas [1], [2], [3], [4]. In a study of African children diagnosed with CM, approximately one quarter were shown to have alternative causes for their neurological syndrome at post-mortem examination [1]. These findings indicate that CM is over-diagnosed, a situation that is likely to have serious consequences for children in whom other treatable or life-threatening conditions are not identified [1], [5]. There is a clear need for a diagnostic test that could distinguish CM from other conditions causing encephalopathy in malaria-endemic areas. In comatose African children, a distinctive retinopathy consisting of haemorrhages, patchy retinal whitening and vessel changes is strongly associated with malaria being the only identifiable cause of death [1].

Features of severe *P. falciparum* malaria include the adhesion of mature parasitized erythrocytes to the microvasculature of vital organs and acute endothelial activation (reviewed in [6]; [7]). Exocytosis of Weibel-Palade bodies (WPBs) occurs in association with endothelial activation and the products of WPBs have been identified as biomarkers of malarial disease severity [8], [9], [10]. WPBs release bioactive products, including von Willebrand factor (VWF), its propeptide (VWFpp), and angiopoietin-2 (Ang-2) into the systemic circulation. Together with vascular endothelial growth factor (VEGF), the angiogenic factors angiopoietin-1 (Ang-1) and Ang-2, are major regulators of the vascular inflammatory response, endothelial activation and endothelial integrity [11], [12]. Ang-1 is constitutively released from perivascular cells including pericytes and smooth muscle cells and signals through the Tie-2 receptor to maintain vascular quiescence and stability. Ang-2 antagonizes Ang-1 function resulting in endothelial activation and increased vascular permeability. Ang-2 sensitizes the endothelium to sub-threshold levels of tumour necrosis factor, resulting in increased expression of adhesion molecules such as ICAM-1 to which parasitized erythrocytes bind [13]. VEGF induces WPB exocytosis, mediates Tie-2 shedding, and regulates Ang-1 and Ang-2 function [11], [14]. Tie-2 is the cognate receptor for Ang-1 and Ang-2 and the soluble form of the receptor, sTie-2, can bind the angiopoietins and regulate their function. WPBs are also an important source of VWF, particularly ultralarge multimers (ULVWF) that are considered biologically hyperactive with respect to their enhanced binding avidity for collagen and platelets [15]. Severe malaria has been associated with increased levels of VWF and ULVWF multimers and decreased levels of the regulatory VWF-specific cleaving protease ADAMTS13 (A disintegrin and metalloprotease with thrombospondin type-1 repeats) [9]. ICAM-1 is a receptor for the cytoadherence of mature parasitized erythrocytes in the cerebral microvasculature and its soluble form (sICAM-1) has been used as a marker of endothelial activation and severe malaria [7], [16], [17]. In addition to the molecular markers and regulators of endothelial quiescence and activation, IP-10, an interferon-gamma inducible chemokine involved in recruitment of activated Th1 cells, has been reported as a biomarker of fatal CM in studies from India and Ghana [18], [19].

Reliable diagnostic and prognostic biomarkers for CM and other forms of severe malaria may improve clinical management, resource allocation and outcome of serious childhood illness. The aim of this study was to evaluate the ability of endothelial biomarkers to discriminate between different clinical disease states in malaria and between cerebral malaria and other conditions associated with fever and altered consciousness in Malawian children. We show that endothelium-based proteins are informative biomarkers of disease severity and clinical response and that a panel of biomarkers can discriminate between retinopathy positive CM and uncomplicated malaria or other CNS infections with a high degree of accuracy. Further, we demonstrate that a distinctive set of endothelium-based proteins is associated with retinopathy in a group of children with coma and parasitaemia.

## Methods

### Ethics Statement

Ethical approval for this study was granted from The College of Medicine Research Committee in Blantyre, Malawi (COMREC) and all parents or guardians gave written informed consent for children to enter the study.

### Study Population

This study was nested within prospective studies examining the pathogenesis and management of CM and central nervous system infections [1], [20], [21]. Convenience samples were selected for children between 1 month and 14 years of age presenting with fever to the Queen Elizabeth Central Hospital (QECH) in Blantyre, Malawi between 1997 and 2009, who met the eligibility criteria for the clinical syndromes and had sufficient plasma samples available. Admission lithium heparin plasma samples were obtained from children after their parents or guardians had given their informed consent. Clinical and demographic data were collected from cases and controls at the time of blood collection, and all subsequent analyses were carried out blind to these details. Efforts were made to minimize bias by selecting subjects from a single population (children presenting to QECH), and by using two types of controls, uncomplicated malaria illness (outpatient) and non-malarial febrile illness with decreased consciousness (inpatient). All participants received standard treatment, including antimalarial and/or antibacterial therapy as indicated, according to Malawian National guidelines.

### Ophthalmological Examination

After admission of a child with altered consciousness, the patient's pupils were dilated by application of drops (tropicamide and phenylephrine) and the fundi were examined by direct and indirect ophthalmoscopy. The findings of an ophthalmologist or experienced clinician were recorded on standardized forms. Retinopathy was defined by the presence of any one or more of the following retinal findings: white-centered haemorrhages, retinal whitening, or vessel changes, with or without papilloedema, as previously described [20], [21]. Papilloedema alone did not constitute retinopathy.

### Definitions of clinical syndromes

#### Cerebral Malaria (CM)

Children meeting the case definition for CM [1], [5] including *P. falciparum* asexual parasitaemia, a Blantyre coma score ≤2 with no improvement following correction of hypoglycemia, and no evidence of an alternative cause for coma including meningitis on examination of cerebrospinal fluid, were eligible for enrolment. Children's fundi were examined and they were classified as retinopathy positive (CM-R) or retinopathy negative (CM-N). CM-R children were considered to be confirmed CM and were used for all analyses comparing clinical groups. Paired admission and 28 day convalescence plasma samples (representing clinical recovery) were collected for each surviving child available for follow-up.

#### Uncomplicated Malaria (UM)

Children were included in the UM group if they presented to the outpatient clinic at Queen Elizabeth Central Hospital, Blantyre, Malawi with febrile illness and a blood film positive for asexual *P. falciparum* without another explanation for fever, and with no malarial complications.

#### CNS controls

Children with non-malarial fever and altered consciousness were included in the study as a further comparator group. Samples were taken from a study looking at suspected central nervous system (CNS) infections (unpublished data). Children with fever or history of fever, a negative malarial smear and at least one of the following: reduced level of consciousness, Blantyre coma score (BCS) ≤4, neck stiffness, photophobia, Kernig's sign, tense fontanelle, focal neurological signs, convulsions, or unexplained irritability in infants, were eligible for enrolment.

### Quantification of Biomarkers

Plasma concentrations of biomarkers Ang-1, Ang-2, sTie-2, VEGF-A, IP-10 and sICAM-1 (DuoSets, R&D Systems, Minneapolis, MN), von Willebrand factor (VWF [capture REF P0226, detection REF A0082]: DAKO, Denmark A/S) and von Willebrand factor propeptide (VWFpp [capture CLB-Pro35, detection CLB-Pro 14.3 HRP conjugated]: Sanquin, Netherlands) were measured by ELISA as follows. According to the manufacturer's instructions, capture antibodies were diluted in PBS (Gibco) overnight at 4°C and were washed with PBS 0.05% Tween 20 (Sigma) five times and blocked for a minimum of 2 hours in PBS 1% BSA (reagent diluent). The samples were then diluted in reagent diluent and standard curves were generated using recombinant proteins (R&D Systems). Normal plasma from a pool of 40 healthy adult Caucasian donors served as a standard for VWFpp and VWF. The plasma pool contained 5.5 nM of VWFpp and 49 nM of VWF. Samples were plated in duplicate and incubated overnight at 4°C, washed five times and detection antibodies were added according to manufacturer recommended dilutions for 2 hours at room temperature (RT). For Ang-1 and Ang-2, the detection antibodies were resuspended one hour prior to use with 2% heat inactivated goat or mouse serum respectively. Following wash steps (7×), VWF and VWFpp were developed using TMB (eBioscience) and the reaction was stopped using 2N H_2_SO_4_. The plate was read at 450 nM (Dynex Technologies Opsys MR plate reader) and concentrations were extrapolated from the standard curve (4-PL) using revelation Quicklink software (v4.04). The ELISA assays from R&D systems were washed (7×) and Extravidin-Alkaline phosphatase (AP) (Sigma) was added 1∶1000 to each well for 1 hour at RT. The plates were then washed a final time (7× in PBS 0.05% Tween 20 and 2× in deionized water) before adding the substrate p-nitrophenyl phosphate (pNPP) (Sigma). The plates were read at 405 nM and concentrations were extrapolated as above.

### Statistics

Data were analyzed in GraphPad Prism v5.0 and SPSS v16.0. All analyses were non-parametric with Spearman's correlation for two-way correlations between biomarkers, Mann-Whitney U tests to compare biomarkers between groups with Holms correction for multiple testing, and receiver operating characteristic (ROC) curves to assess the diagnostic accuracy of the tests. Optimal test thresholds were derived mathematically from the ROC curves using the point on the ROC curve with the lowest value for the formula: (1- sensitivity)^2^ + (1-specificity)^2^. Wilcoxon matched pairs test was used to compare biomarker levels measured at admission and convalescence.

## Results

### Patient Characteristics

A total of 123 febrile children with either UM (n = 32), suspected CNS infections (n = 24), or CM (n = 67) were included in the study. Of the children that met study criteria for CM, 38 were retinopathy positive and were classified as retinopathy-validated CM (CM-R); whereas the other 29 had normal ocular fundi (CM-N) [21]. Children with CM-R were significantly younger than children with clinically defined CM without retinopathy. Demographic and clinical data for these children are shown in Table 1.

**Table 1:** Demographic and clinical characteristics of study population.

	CM		CNS	UM
	CM-R	CM-N		
Number	38	29	24	32
Age, Months	33 (13–82) **	51(14–159)	26 (1–109) <	39 (8–96)
Female, n (%)	17 (44.7)	11 (37.9)	9 (37.5)	17 (53.2)
Presenting BCS	1 (0–2)	1 (0–2)	4 (3–5) +	5
Severe Anaemia, n (%)	28 (73.7%) ***	2 (6.9)	1 (4.5) <	0
Admission Parasitaemia	62,956 (86–1,729,160)	43,631 (36–1,626,240)	0 (0–0) +	#
CSF White Cell Count	0 (0–17)	0 (0–15)	2 (0–6) <	‡
History of Convulsions, n (%)	30 (78.9)	27 (93.1)	8 (44.4)†, <	‡
White Blood Cell Count	9900 (2,800–25,700)	8,800 (1,400–28,000)	‡	‡
Platelet count	45,000 (5,000–262,000)	116,000 (6,000–545,000)	‡	‡
Venous Lactate	6.35 (2.4–16.4)	5.0 (1.6–21.4)	‡	‡

CM: retinopathy positive (CM-R), retinopathy negative (CM-N). Median (range) unless otherwise indicated;

# Clinical information unavailable as children seen on an outpatient basis;

**p<0.01;

***p<0.001 (CM-R vs. CM-N);

†N = 18;

‡ Unavailable;

< p<0.01;

+ p<0.0001 (CM vs. CNS);

BCS: Blantyre Coma Score.

### Endothelial biomarkers differentiate retinopathy positive CM cases from those without retinopathy

**Figure 1:**
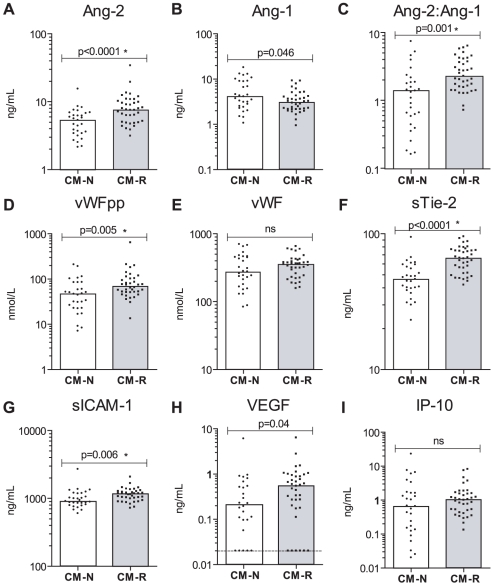
Endothelial biomarkers are associated with retinopathy (CM-R) in children with clinically defined cerebral malaria. Graphs showing the median and scatter of plasma biomarkers (A) Ang-2 (ng/mL), (B) Ang-1(ng/mL), (C) Ang-2:Ang-1, (D) VWF propeptide (VWFpp, mmol/L), (E) VWF (mmol/L), (F) sTie-2 (ng/mL), (G) sICAM-1 (ng/mL), (H) VEGF (ng/mL) and (I) IP-10 (ng/mL) levels in retinopathy negative cerebral malaria (CM-N) or retinopathy positive cerebral malaria (CM-R) as measured by ELISA (Mann-Whitney). ns (not significant, p>0.05), *p<0.05 after Holms correction for multiple comparisons (9 pair-wise comparisons). The limit of detection is represented by a dotted line (VEGF).

Since retinopathy has been established as a discriminant tool in the diagnosis of CM, we examined biomarker levels in children with CM and malaria retinopathy (CM-R) and compared them to children with clinical CM without retinopathy (CM-N). Individually, Ang-2, Ang-2:Ang-1, sTie-2, VWFpp and sICAM-1 were significantly associated with retinopathy (Figure 1, Table 2). For each analyte tested, a receiver operating characteristic (ROC) curve was generated to assess the diagnostic accuracy of the biomarker to discriminate between CM-N and CM-R. The area under the ROC (AUROC) curve was computed and the sensitivity, specificity and positive and negative likelihood ratios were calculated at the optimal biomarker cut-off. sTie-2 and Ang-2 were the best individual predictors of retinopathy with AUROCs of 0.83 (95% CI: 0.73–0.93) and 0.77 (95% CI: 0.65–0.89) respectively.

**Table 2:** Endothelial-biomarkers are associated with retinopathy in a cohort of children with cerebral malaria (CM).

	AUROC CM-R vs CM-N	p-value	Cutoff	Sensitivity (95%CI)	Specificity (95%CI)	Positive likelihood ratio (95% CI)	Negative likelihood ratio (95% CI)
Ang-1	0.64 (0.51–0.78)	0.046	3.2	68 (53–81)	44 (28–62)	1.2 (0.84–1.8)	0.70 (0.38–1.3)
Ang-2	0.77 (0.65–0.89)	<0.0001*	6.2	71 (55–83)	72 (54–85)	2.6 (1.4–4.8)	0.40(0.23–0.69)
Ang-2:Ang-1	0.74 (0.60–0.87)	0.001*	2.0	74 (58–85)	66 (47–80)	2.1 (1.3–3.7)	0.40 (0.22–0.73)
sTie-2	0.83 (0.73–0.93)	<0.0001*	56	74 (58–85)	72 (54–85)	2.7 (1.4–5.0)	0.36 (0.20–0.65)
VWF propeptide	0.71 (0.58–0.85)	0.005*	51	76 (61–87)	62 (44–77)	2.0 (1.2–3.3)	0.38 (0.20–0.72)
VWF	0.58 (0.42–0.73)	0.305	310	68 (53–81)	58 (41–74)	1.7 (1.0–2.7)	0.54 (0.31–0.94)
sICAM-1	0.73 (0.59–0.86)	0.006*	1000	71 (55–83)	69 (51–83)	2.3 (1.3–4.1)	0.42 (0.24–0.73)
VEGF	0.65 (0.52–0.79)	0.040	0.25	74 (58–85)	58 (39–74)	1.7 (1.1–2.8)	0.46(0.24–0.85)
IP-10	0.58 (0.43–0.73)	0.312	0.71	66 (50–79)	54 (36–70)	1.4 (0.90–2.2)	0.64 (0.37–1.1)

Median (range), Mann-Whitney U test;

*p<0.05 after Holms correction for 9 pair-wise comparisons;

CM-R: Cerebral malaria, retinopathy positive

CM-N: Cerebral malaria, retinopathy negative, AUROC: Area under the receiver operating characteristic curve.

### Endothelial biomarkers differ between CM and UM

**Figure 2:**
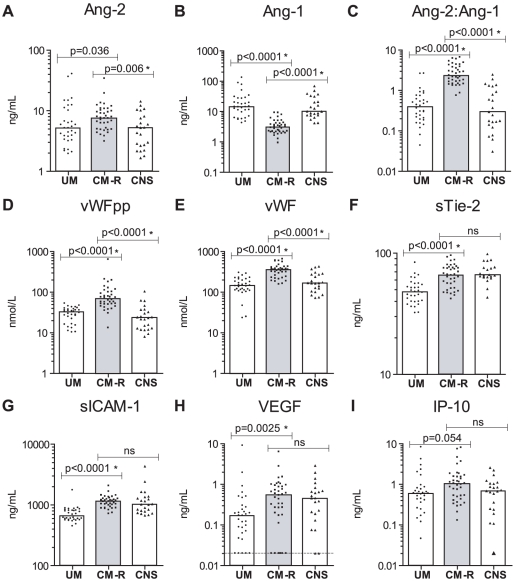
Endothelial biomarkers differentiate retinopathy validated cerebral malaria (CM-R) from uncomplicated malaria (UM) and children with fever and decreased consciousness due to other causes (CNS). Graphs showing the median and scatter of plasma biomarkers (A) Ang-2 (ng/mL), (B) Ang-1(ng/mL), (C) Ang-2:Ang-1, (D) VWF propeptide (VWFpp, mmol/L), (E) VWF (mmol/L), (F) sTie-2 (ng/mL), (G) sICAM-1 (ng/mL), (H) VEGF (ng/mL) and (I) IP-10 (ng/mL) levels in UM, non-malarial febrile illness with decreased consciousness (CNS) or retinopathy validated cerebral malaria (CM-R) as measured by ELISA (Mann-Whitney). ns (not significant, p>0.05), *p<0.05 after Holms correction for multiple comparisons (9 pair-wise comparisons). The limit of detection is represented by a dotted line (VEGF).

We compared admission levels of plasma biomarkers in children with CM-R (n = 38) to children with UM (n = 32). The median concentration of Ang-1 was significantly lower, and median levels of Ang-2:Ang-1, sTie-2, VWFpp, VWF, sICAM, VEGF were significantly higher in patients with CM-R than in patients with UM (Figure 2, Table 3).

**Table 3:** Biomarker levels in Malawian children with uncomplicated malaria (UM), retinopathy confirmed cerebral malaria (CM-R) or non-malarial fever with altered consciousness (CNS).

	UM (n = 32)	CM-R (n = 38)	CNS (n = 24)
Ang-1, ng/mL	14 (4.2–132)**	3.1 (0.94–9.2)	10 (3.1–69)**
Ang-2, ng/mL	5.2 (2.0–41)	7.6 (3.1–34)	5.08 (1.67–14.29)*
Ang-2:Ang-1	0.39 (0.04–2.6)**	2.3 (0.75–6.6)	0.34 (0.03–2.5)**
sTie-2, ng/mL	48 (32–84)**	66(42–95)	67 (43–124)
VWFpp, nmol/L	32 (10–53)**	70 (13–1127)	23 (8.1–106)**
VWF, nmol/L	144 (23–301)**	357 (157–654)	169 (73–524)**
sICAM-1, ng/mL	660 (447–1761)**	1154 (718–2073)	1016 (675–4358)
VEGF, ng/mL	0.17 (0.02–9.1)*	0.55 (0.02–6.4)	0.46 (0.02–2.9)
IP-10, ng/mL	0.60 (0.05–8.3)	1.0 (0.13–8.1)	0.69 (0.02–2.6)

Median (range);

*p<0.05;

**p<0.01 for difference compared to CM-R (corrected for multiple testing at 18 pair-wise comparisons using Holm's test).

ROC curves were generated to assess the diagnostic accuracy of the biomarkers to discriminate between UM and CM-R. The AUROC curve was computed and the sensitivity, specificity and positive and negative likelihood ratios were calculated at the optimal biomarker cut-off (Table 4). Ang-1, sTie-2, VWFpp, VWF, ICAM, VEGF were each able to differentiate between UM and CM-R whereas IP-10 was not. Although Ang-2 on its own was no longer significant after correcting for multiple comparisons, the Ang-2:Ang-1 ratio had an AUROC as good as Ang-1 and resulted in an improved positive likelihood ratio (LR(+)) versus Ang-1 alone [Ang-1 LR(+) = 7.1 compared to Ang-2:Ang-1 LR(+) = 18].

**Table 4:** Receiver operating characteristic curves of endothelial biomarkers in children with uncomplicated malaria (UM) or cerebral malaria with retinopathy (CM-R).

	AUROC UM vs. CM-R (95% CI)	p-value	Cutoff	Sensitivity (95%CI)	Specificity (95%CI)	Positive likelihood ratio (95%CI)	Negative likelihood ratio (95%CI)
Ang-1	0.96 (0.93–1.0)	<0.0001*	5.3	94 (80–98)	87 (73–94)	7.1 (3.1–16)	0.072 (0.019–0.28)
Ang-2	0.65 (0.51–0.79)	0.0359	6.1	63 (45–77)	71 (55–83)	2.2 (1.2–3.8)	0.53 (0.32–0.86)
Ang-2: Ang-1	0.96 (0.93–1.0)	<0.0001*	0.95	94 (80–98)	95 (83–99)	18 (4.6–69)	0.066 (0.017–0.25)
sTie-2	0.82 (0.72–0.92)	<0.0001*	56	78 (61–89)	74 (58–85)	3.0 (1.7–5.2)	0.30 (0.15–0.59)
VWF propeptide	0.93 (0.87–0.99)	<0.0001*	44	91 (76–97)	84 (70–93)	5.7 (2.7–12)	0.11 (0.038–0.33)
VWF	0.93 (0.88–0.99)	<0.0001*	220	81 (65–91)	84 (70–93)	5.1 (2.4–11)	0.22 (0.11–0.46)
sICAM-1	0.94 (0.87–1.0)	<0.0001*	870	91 (76–97)	92 (79–97)	11 (3.9–34)	0.10 (0.035–0.30)
VEGF	0.71 (0.58–0.84)	0.0025*	0.25	72 (55–84)	74 (58–85)	2.7 (1.5–4.9)	0.38 (0.21–0.69)
IP-10	0.64 (0.50–0.77)	0.0504	0.72	69 (51–82)	66 (49–79)	2.0 (1.2–3.3)	0.48 (0.27–0.83)

Median (range), Mann-Whitney U test;

*p<0.05 after Holms correction for 9 pair-wise comparisons;

CM-R: Cerebral malaria, retinopathy positive

UM: Uncomplicated malaria, AUROC: Area under the receiver operating characteristic curve.

### Distinct biomarker profiles in CM differ between retinopathy validated CM and other causes of fever and altered mental status

Distinguishing CM-R from other causes of fever and altered level of consciousness is clinically challenging yet critical for instituting timely, specific, and potentially life-saving treatment. We hypothesized that CM-R is associated with a characteristic pattern of endothelial biomarker abnormalities, which may be clinically informative in distinguishing CM-R from other causes of fever and altered consciousness. Comparing children with CM-R (n = 38) to a control group of children admitted with non-malarial febrile illness and altered level of consciousness (CNS) (n = 24), median Ang-1 levels were lower, and median Ang-2, Ang-2:Ang-1, VWFpp and VWF higher in children with CM-R compared to CNS controls (Figure 2, Table 3).

Using ROC curve analysis, three biomarkers, Ang-1, VWFpp, and VWF, discriminated children with CM-R from children with other suspected CNS infections (Table 5). Median Ang-2 was elevated in CM-R compared to the CNS controls, and the Ang-2:Ang-1 ratio had an AUROC equal to that of Ang-1 alone but with a two-fold increase in the positive likelihood ratio (Table 5). sICAM-1, sTie-2, and VEGF, while useful biomarkers for UM vs. CM-R, did not discriminate between CM-R vs. CNS (Table 5).

**Table 5:** Receiver operating characteristic curves of endothelial biomarkers in children with fever and altered consciousness (CNS) or cerebral malaria with retinopathy (CM-R).

	AUROC CNS vs. CM-R	p-value	Cutoff	Sensitivity (95%CI)	Specificity (95%CI)	Positive likelihood ratio (95%CI)	Negative likelihood ratio (95%CI)
Ang-1	0.93 (0.88–0.99)	<0.0001*	5.3	88 (69–96)	87 (73–94)	6.7 (2.9–15)	0.14 (0.05–0.42)
Ang-2	0.71 (0.55–0.84)	0.006*	6.3	67 (47–82)	71 (55–83)	2.3(1.3–4.1)	0.47 (0.26–0.86)
Ang-2: Ang-1	0.93 (0.87–0.99)	<0.0001*	1.4	71 (51–85)	95 (83–99)	13 (3.4–53)	0.31 (0.16–0.58)
sTie-2	0.62 (0.49–0.78)	0.1087	66	8.3 (2.3–26)	74 (58–85)	1.2 (0.99–1.6)	0.32 (0.076–1.3)
VWF propeptide	0.89(0.80–0.98)	<0.0001*	38	79 (60–91)	84 (70–93)	5.0 (2.3–11)	0.25 (0.11–0.55)
VWF	0.80 (0.66–0.92)	<0.0001*	310	63 (43–79)	84 (70–93)	4.0 (1.8–8.8)	0.45 (0.26–0.76)
sICAM-1	0.59 (0.41–0.74)	0.254	1000	38 (21–57)	92 (79–97)	4.8 (1.4–16)	0.68 (0.49–0.94)
VEGF	0.54 (0.39–0.69)	0.587	0.53	39 (22–59)	74 (58–85)	1.5 (0.71–3.1)	0.83 (0.57–1.2)
IP-10	0.64 (0.50–0.78)	0.0673	0.91	52 (33–71)	66 (50–79)	1.5 (0.85–2.8)	0.73 (0.45–1.2)

Median (range), Mann-Whitney U test;

*p<0.05 after Holms correction for 9 pair-wise comparisons;

CM-R: Cerebral malaria, retinopathy positive.

CNS: children with non-malarial fever with decreased consciousness, AUROC: Area under the receiver operating characteristic curve.

### Correlation between endothelial biomarkers

Complex interactions have been reported between molecular regulators of endothelial function [12]. Therefore we postulated that significant correlations would exist between endothelial biomarkers. After applying two-way rank correlations we found significant correlations between Ang-2 and sTie-2, VWFpp, VWF, sICAM-1, and IP-10 and inverse correlations between Ang-1, Ang-2, VWF, VWFpp, sICAM-1 and IP-10 (Table 6). Most of the endothelial biomarkers were significantly associated with each other, except for VEGF, which was only associated with IP-10.

**Table 6:** Two-way rank correlations between biomarkers.

	Ang-2	Tie-2	VWFpp	VWF	sICAM-1	VEGF	IP-10
Ang-1	−0.21**	−0.10	−0.55**	-0.63**	−0.51**	0.034	−0.18*
Ang-2		0.33**	0.55**	0.42**	0.36**	0.12	0.54**
sTie-2			0.251**	0.25**	0.44**	0.17	0.13
VWFpp				0.80**	0.58**	0.055	0.57**
VWF					0.60**	0.14	0.37**
sICAM-1						0.16	0.43**
VEGF							0.25**

Spearman's rho;

*p<0.05;

**p<0.01;

n = 123.

### Endothelial biomarker levels are associated with clinical recovery

**Figure 3:**
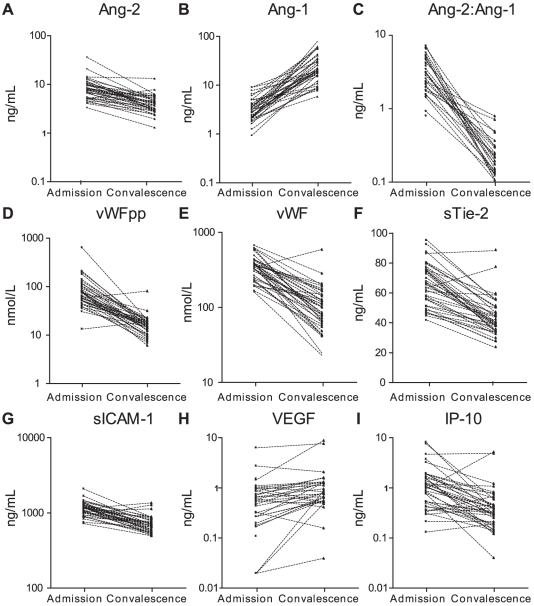
Biomarker levels at admission and follow up. Plasma levels of biomarkers were measured at admission and 28 days post-treatment in a cohort of retinopathy positive children with cerebral malaria. Wilcoxon signed rank test with Holms correction (9 pair-wise comparisons) was used to compare levels of (A) Ang-2 (ng/mL); sum of signed ranks (W), (W, p-value: 746, p<0.0009); (B) Ang-1 (ng/mL), (W, p-value: −741, p<0.0009); (C) Ang-2:Ang-1, (W, p-value: 741, p<0.0009); (D) VWF propeptide (nM), (W, p-value: 768, p<0.0009); (E) VWF (nM), (W, p-value: 740, p<0.0009); (F) sTie-2 (ng/mL) (W, p-value: 754, p<0.0009); (G) sICAM-1 (ng/mL), (W, p-value: 732, p<0.0009); (H) VEGF (ng/mL), (W, p-value: −388, p = 0.001); and (I) IP-10 (ng/mL), (W, p-value: 607, p<0.0009).

We hypothesized that sequential measurements of endothelial biomarker levels could provide objective and quantitative evidence of clinical recovery and disease resolution. To test this hypothesis, we obtained paired measurements of endothelial biomarkers at admission and at day 28, following treatment and recovery, from 38 survivors of CM-R. Levels of Ang-1 displayed a uniform and consistent increase in all participants, whereas levels of Ang-2, Tie-2, VWFpp, VWF, sICAM-1, and IP-10 decreased with convalescence (Figure 3, Table S1). Overall, VEGF showed a significant increase in levels at convalescence (Table S1). Notably, the Ang-2:Ang-1 ratio showed the most dynamic range between levels at admission and follow-up and there was a universal and consistent decrease in Ang-2:Ang-1 levels associated with clinical recovery.

## Discussion

The diagnosis of cerebral malaria in children is clinically challenging since the syndrome may be confused with other causes of fever and altered consciousness. Diagnostic tools that could accurately identify children with “true” CM would enable improved triage and management of these life-threatening infections. Currently retinopathy is the best tool to predict which febrile, comatose children have true CM [1], [20]–[22], but it has operational constraints. Alternative methods of discriminating between these groups have not been described. Identifying retinopathy in a comatose child greatly increases the confidence with which the clinical syndrome can be attributed to malaria. Direct ophthalmoscopy through dilated pupils can be used to observe retinal changes, but a more accurate picture is provided by indirect ophthalmoscopy, a procedure that is usually not available where resources are limited. In this study, we tested the hypothesis that plasma biomarkers may function as surrogate markers for malarial retinopathy. We show that several biomarkers (Ang-2, sTie-2, VWFpp and sICAM-1) were significantly associated with retinopathy in children with clinical defined CM (Fig. 1, Table 2). We report that a panel of endothelial and angiogenic biomarkers is able to discriminate, with a high degree of accuracy, children with retinopathy-confirmed CM (CM-R) from those with uncomplicated infection (UM) or with non-malarial febrile illness and altered consciousness (CNS controls) (Fig. 2. Table 3&4). This is the first study to demonstrate the specificity of Ang-1 for CM in children, and we confirm and extend previous observations of elevated levels of VWFpp and VWF in children with severe malaria compared to non-malarial febrile illness [8]. Further, we show a marked and uniform decrease in Ang-2:Ang-1 at follow-up (Fig. 3), suggesting that the ratio between these two proteins may offer an approach to monitor clinical response. This study represents a significant extension to previous studies of endothelial biomarkers in malaria as it includes a retinopathy-confirmed group of children with cerebral malaria and also includes children with decreased consciousness due to other febrile illnesses. Finally, we are able to go beyond description to examine different putative causal biomarkers of endothelial activation and postulate on their role in disease pathogenesis and clinical recovery.

The endothelium is a dynamic organ system representing the interface between the vascular space and vital organs. The regulation of endothelial integrity is of critical importance, particularly in the face of infection-related injury. Endothelial adhesion of parasitized red cells and endothelial activation are prominent features in the pathology of fatal malaria. Parasitized erythrocytes bind to the endothelium directly through endothelial receptors and may indirectly bind through VWF and platelet complexes [23]. There is evidence to suggest that blood-brain-barrier dysfunction and breakdown occurs in paediatric CM [24]–[26]. However, the pathophysiology of CM is poorly understood. A detailed understanding of endothelial activation and regulation during infection may provide new insights into the molecular basis of severe and fatal malaria.

Angiopoietins are critical regulators of endothelial activation and integrity. Elevated Ang-2 levels have previously been associated with severe malaria in paediatric and adult populations and strategies to block Ang-2 have been suggested as novel interventions for severe malaria [10], [27], [28]. In this study, Ang-2 was elevated in CM-R compared to UM and CNS controls but became of borderline significance after correcting for multiple comparisons. However, inclusion of Ang-2 as a component of the Ang2:Ang-1 ratio markedly improved the specificity and positive likelihood ratio compared to Ang-1 or Ang-2 alone. Levels of Ang-2 observed in this study were lower than those reported in Ugandan children with CM [27]. This difference is likely attributable to the present study design that excluded fatal cases, in which Ang-2 levels are highest. Ang-2 has been associated with increased disease severity [10], [28] and increased risk of death [10] in Asian adults with severe malaria. Together, these data suggest that changes in Ang-2 are reflective of disease severity and mortality and may be a good surrogate endpoint for trials investigating mortality or evaluating adjunctive therapies. In the context of endothelial biology, the balance between Ang-1 and Ang-2 regulates the functional responsiveness of the endothelium. Ang-1 is synthesized by peri-endothelial cells to promote vascular quiescence under normal physiologic conditions; however, the release of Ang-2 from WP bodies can inhibit Ang-1 signalling in a dose-dependent manner, resulting in local destabilization of the endothelium [29]. In this study, there were markedly lower Ang-1 levels at presentation in children with CM-R compared to those with UM or the CNS controls. The observed decreases in Ang-1 levels combined with increases in Ang-2 may contribute to the endothelial dysfunction observed in CM.

The functions of Ang-1 and Ang-2 are also modulated by interactions with VEGF. Under normal physiological conditions of high Ang-1 and low Ang-2, VEGF can stabilize the endothelium in an anti-apoptotic state and be neuroprotective [30], [31]. However, when Ang-1 levels are low, VEGF can act on the endothelium unopposed, resulting in the upregulation of Ang-2 mRNA, exocytosis of WP bodies, increased permeability of endothelial cells and upregulation of tissue factor and ICAM-1[32]–[36]. This may be particularly important in the context of paediatric CM, where coagulopathy and increased tissue factor expression may occur and Ang-1 levels are low [37], [38]. In this study, VEGF was elevated in children with retinopathy confirmed CM compared to children with uncomplicated malaria. These findings are in contrast to reports examining VEGF in severe malaria in adults from Southeast Asia, which have reported a decrease in VEGF in fatal CM cases [19] and decreased VEGF associated with increased disease severity [10]. These studies postulate that VEGF may be neuroprotective and associated with wound healing [19], or may reflect the accumulation of VEGF within the parasitophorous vacuole [10], [39]. Other reports from children have shown no difference in CSF levels of VEGF between deaths due to malaria vs. those attributed to other causes [18]. However, a study of Kenyan children with CM showed plasma VEGF levels were positively correlated with TNF and inversely correlated with a neuroprotective agent, erythropoietin, and high levels of plasma VEGF were associated with an increased risk of seizures, raised intracranial pressure, and papilloedema [40]. These differences may be due to geographic location (Africa vs. Asia) or patient population (pediatric vs. adult).

Other studies in pediatric populations also suggest that VEGF is associated with disease severity. A study looking at VEGF levels in children with sepsis and meningococcemia reported the highest VEGF levels in patients with septic shock [41] and elevated VEGF was a useful prognostic indicator in Kawasaki disease, an acute febrile vasculitis in children [42]. In our study, despite being elevated in CM-R, VEGF was not a particularly informative marker, as it was variable between admission and follow up in children with CM, and was non-specifically elevated in the CNS controls. However, VEGF may be playing an under-appreciated role in endothelial regulation based on the presence or absence of Ang-1 or Ang-2 in the local milieu. Consequently, it may be premature to rule out VEGF as a mediator of severe malaria, given its diverse functions. It is worth noting that VEGF is contained primarily within platelets, and plasma levels can be affected by differences in sample processing (anti-coagulant used, centrifugation time and speed, etc.). Together, our VEGF data supports the idea that VEGF may be associated with disease severity and neurologic complications in CM; however, due to its local action, future studies are warranted to further elucidate the role of VEGF in disease severity.

ICAM-1 is upregulated in the cerebral endothelium during malaria infection and is associated with parasite sequestration within the cerebral vasculature, a pathological hallmark of CM [17]. sICAM-1 is released by activated endothelium during malaria and has been reported as a biomarker of disease severity [16], [43]. Similarly, in our study, sICAM-1 was able to discriminate between UM and CM, but it was also elevated in the CNS control group. In this population, IP-10 was non-significantly elevated in retinopathy-confirmed CM and displayed poor performance as a biomarker. Previous reports have identified IP-10 as a good prognostic biomarker for malaria mortality [19]; however, this could not be assessed in the current study where fatal cases were excluded. Recent reports have described increased circulating concentrations of VWF and activation of the coagulation system in severe malaria, with possible implications for pathogenesis [8], [9], [37]. It has been postulated that an increase in ultra large VWF strings and a decrease in its cleavage protein ADAMTS13 may result in platelet accumulation and contribute to sequestration of parasitized erythrocytes [23]. In the current study, both VWF and VWFpp were elevated in CM-R compared to UM or CNS controls, suggesting that these proteins are also good candidate biomarkers for CM.

The endothelial proteins measured in this study returned to a normal range in association with clinical recovery, suggesting that the alterations in biomarker levels at presentation were mediated by the infection status of the child rather than a natural host-mediated susceptibility as a result of genetic or epigenetic changes in the biomarkers examined. In previous studies, VWFpp was shown to return to baseline three days after the initiation of anti-malarial therapy [8], and Ang-2 and the RH-PAT index, a measure of peripheral endothelial dysfunction, returned to normal four days post treatment [10].

In summary, our study is the first to examine a panel of endothelial-based biomarkers in a well characterized patient population. Recent investigations have moved towards the inclusion of non-malaria febrile illness (NMFI) as a control group to investigate the specificity of markers for malaria infection. In this study, we included a NMFI group with decreased consciousness, which approximates the clinically ideal control group, i.e. children with incidental parasitemia and coma (bacterial meningitis, viral encephalopathy, etc.). CM is a clinical definition based on the presence of malaria parasitemia and coma with no other identifiable cause. However, in malaria endemic areas, there are often resource constraints that make exclusion of alternative causes of coma problematic.

The results of this study are encouraging, and suggest that a limited panel of endothelial biomarkers may be useful in differentiating between retinopathy positive CM and non-malarial febrile illness with decreased consciousness. However, these results will need to be confirmed in prospective studies and assessed alongside other infectious causes of coma in paediatric populations. Further validation of biomarkers needs to be performed to determine whether these markers: i) are informative in stratifying subjects in clinical trials or pathogenesis studies; ii) may have clinical utility in the diagnosis of true CM, assessment of disease severity and response to therapy (allocation of resources), or to determine prognosis; and iii) represent novel therapeutic targets for adjunctive therapy.

## Supporting Information

Table S1 Levels of plasma biomarkers at admission and one month follow up visit from retinopathy positive children with CM.
(DOC)
