# Supplementary material for: Endothelium-Based Biomarkers Are Associated with Cerebral Malaria in Malawian Children: A Retrospective Case-Control Study
Source: PLoS One. 2010 Dec 29;5(12):e15291. doi: 10.1371/journal.pone.0015291 (PMC3012131; doi:10.1371/journal.pone.0015291)
Supplement: Table S1 — Levels of plasma biomarkers at admission and one month follow up visit from retinopathy positive children with CM. (DOC) [file pone.0015291.s001.doc]

**Supplementary Table 1. Levels of plasma biomarkers at admission and one month follow up visit** from retinopathy positive children with CM.

|  | **Admission** | **Convalescence** | **p-value** | **Corrected p-value*** |
| --- | --- | --- | --- | --- |
| Ang-1 | 3.1 (0.94-9.2) | 21 (6.0-100) | <0.0001 | <0.0009 |
| Ang-2 | 7.5 (3.1-34) | 3.8 (1.2-13) | <0.0001 | <0.0009 |
| Ang-2:Ang-1 | 0.95 (0.03-6.6) | 0.15 (0.03-0.77) | <0.0001 | <0.0009 |
| sTie-2 | 66 (42-95) | 40 (24-88) | <0.0001 | <0.0009 |
| VWFpp | 74 (13-1127) | 16 (6.1-122) | <0.0001 | <0.0009 |
| VWF | 353 (157-654) | 98 (23-579) | <0.0001 | <0.0009 |
| sICAM-1 | 1164 (718-2073) | 687 (494-1356) | <0.0001 | <0.0009 |
| VEGF | 0.54 (0.02-6.4) | 0.80 (0.04-9.0) | 0.001 | 0.001 |
| IP-10 | 1.1 (0.13-8.1) | 0.33 (0.04-5.2) | <0.0001 | <0.0009 |

Median (range)

* Wilcoxon signed rank test with Holms correction (9 pair-wise comparisons)
